# Classification of Non-Small Cell Lung Cancer Based on Copy Number Alterations

**DOI:** 10.1371/journal.pone.0088300

**Published:** 2014-02-05

**Authors:** Bi-Qing Li, Jin You, Tao Huang, Yu-Dong Cai

**Affiliations:** 1 Institute of Systems Biology, Shanghai University, Shanghai, P.R. China; 2 Key Laboratory of Systems Biology, Shanghai Institutes for Biological Sciences, Chinese Academy of Sciences, Shanghai, P. R. China; 3 The Key Laboratory of Stem Cell Biology, Institute of Health Sciences, Shanghai Institutes for Biological Sciences, Chinese Academy of Sciences, Shanghai, P. R. China; 4 Department of Genetics and Genomic Sciences, Mount Sinai School of Medicine, New York City, New York, United States of America; UNIVERSITY MAGNA GRAECIA, Italy

## Abstract

Lung cancer is one of the leading causes of cancer mortality worldwide and non–small cell lung cancer (NSCLC) accounts for the most part. NSCLC can be further divided into adenocarcinoma (ACA) and squamous cell carcinoma (SCC). It is of great value to distinguish these two subgroups clinically. In this study, we compared the genome-wide copy number alterations (CNAs) patterns of 208 early stage ACA and 93 early stage SCC tumor samples. As a result, 266 CNA probes stood out for better discrimination of ACA and SCC. It was revealed that the genes corresponding to these 266 probes were enriched in lung cancer related pathways and enriched in the chromosome regions where CNA usually occur in lung cancer. This study sheds lights on the CNA study of NSCLC and provides some insights on the epigenetic of NSCLC.

## Introduction

Lung cancer is one of the leading cause of cancer mortality worldwide [1]. Basing on the 2011 International Association for the Study of Lung Cancer/American Thoracic Society/European Respiratory Society (IASLC/ATS/ERS) lung adenocarcinoma classification, it is now classified into 5 different subtypes: Atypical adenomatous hyperplasia (AAH), Adenocarcinoma in situ (AIS) (nonmucinous, mucinous, or mixed nonmucinous/mucinous), Minimally invasive adenocarcinoma (MIA) (≤3 cm lepidic predominant tumor with ≤5 mm invasion), Invasive adenocarcinoma, and variants of invasive adenocarcinoma, and each of them has its own histological feature [2]. Non–small cell lung cancer (NSCLC) accounts for 85% of all lung cancers. The most frequent histologic subtypes of NSCC is adenocarcinoma (ACA) and squamous cell carcinoma (SCC), accounting for 50% and 30% of NSCLC cases, respectively [3]. ACA is the most common histologic subtype reported with lung cancer in the never smokers (LCINS) [4], which is a cancer of an epithelium which originates in glandular tissue. SCC is a cancer of squamous epithelial cell, which arises most often in segmental bronchi and related to lobar and main stem bronchus occurs by its extension [5], and its incidence is correlated with smoking period [6] compared with ACA. Historically, well differentiated SCC cells include the morphologic features such as intercellular bridging, squamous pearl formation and individual cell keratinization [5]. Nowadays, medicine development in NSCLC has introduced histologic subtyping, the differentiation of ACA from SCC in biopsy specimens, as an important factor for effective treatment choice and molecular therapy target. For example pemetrexed, antifolate agent, is effective in the treatment of patients with non-squamous NSCLC but should not be recommended for the treatment of squamous cell carcinoma [7]. Bevacizumab, combined with paclitaxel/carboplatin, has excessive toxic effects in squamous-cell carcinoma [8], while it could significantly increase overall survival rate of patients with cancers of non-squamous histology [9], [10]. Traditional diagnosis method to distinguish adenocarcinoma from squamous cell carcinoma, is based on the histologic section and patients' smoking habit. However, because of the individual heterogeneity of lung cancer, this method cannot correctly distinguish ACA and SCC in some cases efficiently. Recently, immunohistochemistry is being used in biopsy and cytology material [11] as a complement, and several genes have been discovered as the immunohistochemical marker. Kargi et al. found thyroid transcription factor-1 (TTF-1) is a marker in immunostaining for ACA, while p63 and cytokeratins (CK) 5/6 are marks for SCC [12]. Moreover, molecular targeted therapy has been more and more used in NSCLC as the promising treatment strategy in recent years. It is demonstrated that superior efficacy of tyrosine kinase inhibitors (TKIs) as compared to standard chemotherapy for patients with EGFR-mutant tumors [13]. Kwak et al. also explored the small-molecule inhibitor of the ALK tyrosine kinase could be used as the efficacious therapy in advanced ALK-positive tumors in an early-phase clinical trial [14]. Therefore, it is meaningful to identifying genes which have distinct genetics features in ACA and SCC that could be used as prognostic factor or potential target for medical therapy.

Previous analysis has showed CNAs are common in almost all human cancers [15], [16]. In NSCLC, CNAs increase with disease progression and CNAs are both positionally and functionally clustered [17]. Furthermore, Giovanni Tonon el at. found despite their distinct histopathological phenotypes, ACA and SCC genomic profiles showed a nearly complete overlap, with only one clear SCC-specific amplicon on 3q26–29 [18].

In this study, to figure out the key genes distinguishing ACA and SCC from each other, we compare the genome-wide copy number alterations (CNAs) patterns of 208 early stage ACA and 93 early stage SCC tumor samples. By means of the feature selection and analysis methods, including the Maximum Relevance Minimum Redundancy method (mRMR) and the Incremental Feature Selection (IFS) method, 266 optimal CNA probes were selected for the discrimination of ACA and SCC. The classification model was built with Nearest Neighbor Algorithm (NNA). As a result, the classifier achieved a overall MCC of 0.6616. Further analysis on the 266 CNA related genes showed that they were closely associated with lung cancer.

## Materials and Methods

### Dataset

We used the copy number alterations data from the non-small cell lung cancer study of Huang et al. [19]. In their study, a series of 301 snap-frozen tumor samples from NSCLC patients was collected during surgery or biopsy from the Massachusetts General Hospital (MGH), Boston, MA and the National Institute of Occupational Health, Oslo, Norway. The clinical information of these 301 samples was given in File S1. The copy number profiling of 208 early stage adenocarcinoma tumors (ACA) samples and 93 early stage squamous cell carcinoma tumors (SCC) were retrieved from NCBI Gene Expression Omnibus (GEO) with the accession number of GSE34140. The copy number profile was obtained using the using Affymetrix 250 K Nsp GeneChip. Only 256,554 probes on somatic chromosomes were analyzed. The SNP probes were mapped to the RefSeq genes with 2 kb extension both upstream and downstream using the UCSC Genome Browser. Among the 256,554 probes on somatic chromosomes, 104,256 probes were mapped to 11,700 genes [19].

### mRMR method

We used Maximum Relevance Minimum Redundancy (mRMR) method to rank the importance of the probes [20]. mRMR method could rank probes based on both their relevance to the class of samples and the redundancy among probes. A smaller index of a probe denotes that it has a better trade-off between maximum relevance to class of samples and minimum redundancy.

Both relevance and redundancy were quantified by mutual information (MI), which estimates how much one vector is related to another. The MI equation was defined as below:
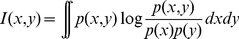
(1)


In equation (1), 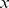, 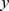 are vectors, 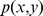 is their joint probabilistic density, and 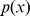 and 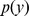 are the marginal probabilistic densities.

Let 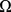 denote the whole probe set, 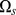 denote the already-selected probe set containing *m* probes and 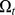 denote the to-be-selected probe set containing *n* probes. The relevance 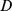 between a probe 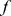 in 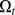 and the class of sample 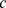 can be calculated by:
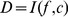
(2)


The redundancy 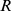 between a probe 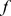 in 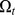 and all the probes in 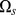 can be calculated by:
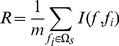
(3)


To get the probe 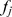 in 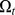 with maximum relevance and minimum redundancy, the mRMR function combines equation (2) and equation (3) and is defined as below:
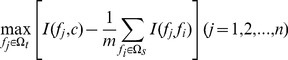
(4)


The mRMR probe rating would be executed N rounds when given a probe set with N (N = m+n) probes. After N rounds of execution, a probe set 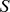 is produced:
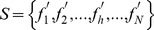
(5)


In 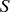, index *h* indicates at which round that the probe is selected. The smaller the index *h* is, the earlier the probe satisfies equation (4) and the better the probe is.

### Nearest neighbor algorithm (NNA)

Nearest Neighbor Algorithm (NNA) [21], [22], which has been widely used in bioinformatics and computational biology [23], [24], [25], [26], [27], was adopted to predict the class of samples. The “nearness” was calculated according to the following equation
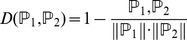
(6)where 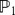 and 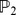
are two vectors representing two samples, 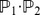 is their dot product, 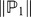 and 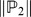 are their moduluses. The smaller the 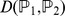, the more similar the two samples are.

For an intuitive illustration of how NNA works, see Fig.5 of [28].

### Jackknife Cross-Validation Method

Jackknife Cross-Validation Method [23], [24], [29], [30] (also called the Leave-one-out cross-validation, LOOCV) was used to evaluate the performance of a classifier. In Jackknife Cross-Validation Method, every sample is tested by the predictor that is trained with all the other samples. Let TP denotes true positive. TN denotes true negative. FP denotes false positive and FN denotes false negative. To evaluate the performance of our predictor, the prediction accuracy, specificity, sensitivity and MCC (Matthews's correlation coefficient) were calculated as below:
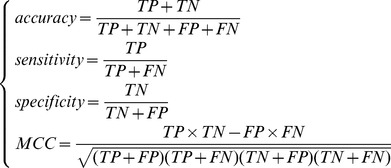
(7)


### Incremental Feature Selection (IFS)

Based on the ranked probes rated by mRMR evaluation, we used Incremental Feature Selection (IFS) [31], [32], [33] to determine the optimal number of probes. During IFS procedure, probes in the ranked probe set are added one by one from higher to lower rank. A new probe set is composed when one probe is added. Thus N probe sets would be composed given N ranked probes. The i-th probe set is:
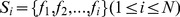
(8)


For each of the N probe sets, an NNA predictor was constructed and tested using LOOCV. With N prediction accuracies, sensitivities, specificities and MCCs calculated, we obtain an IFS table with one column being the index i and the other columns to be the prediction accuracy, sensitivity, specificity and MCC. The optimal probe set (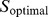) is the one, using which the predictor achieves the best prediction performance.

### Functional enrichment analysis of CNAs genes

Functional annotation tool of GATHER [34] was used for KEGG pathway, GO and chromosome region enrichment analysis. All the genes in the human genome were selected as background during the enrichment analysis.

## Results and Discussion

### The mRMR Result

Listed in the File S2 are two kinds of outcomes obtained by running the mRMR software: one is called the “MaxRel feature list” that ranked all the probes according to their relevance to the class of samples; the other one is the “mRMR feature list” that ranked the probes according to the criteria of maximum relevance and minimum redundancy. In the mRMR probe list, the smaller the index of a probe was, the more important the probe would be for the discrimination of two kinds of NSCLC. Accordingly, the mRMR feature list could be used to establish the optimal feature set in the IFS procedure.

### IFS and Final Optimal Feature Set

**Figure 1:**
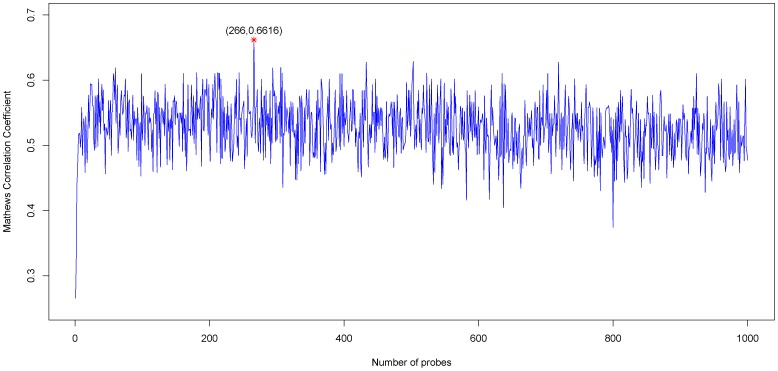
IFS curve for the adenocarcinoma (ACA) and squamous cell carcinoma (SCC) samples classification. The IFS curves were drawn based on the data in File S3. The MCC reached the peak when the number of probes was 266. The 266 probes thus obtained were used to compose the optimal probe set for discrimination of adenocarcinoma (ACA) and squamous cell carcinoma (SCC).

**Figure 2:**
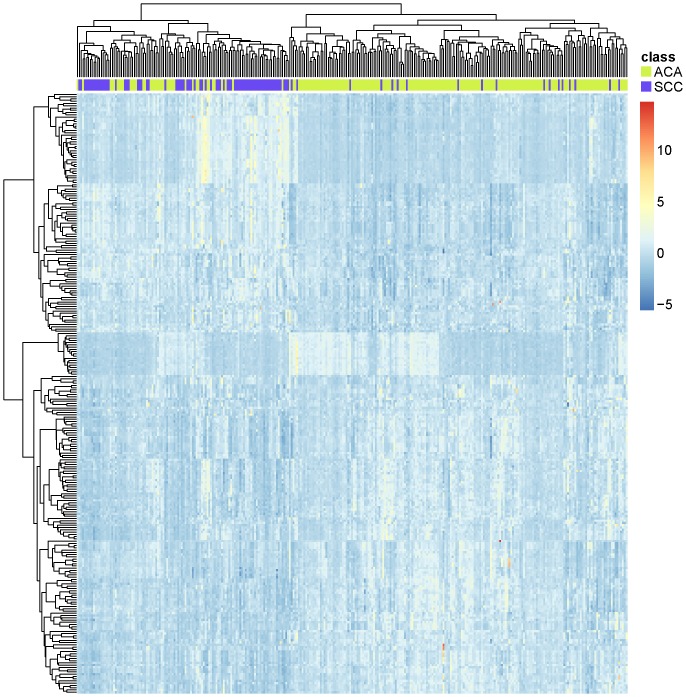
Heatmapof 208 adenocarcinoma (ACA) samples and 93 squamous cell carcinoma (SCC) samples with 266 selected probes. Samples are arranged along the X axis and probes along the Y axis. Each square represents the copy number of a given probe in an individual sample. Red is increased copy number and blue is decreased copy number relative to the mean- and sample-centered scaled copy number across the samples. Adenocarcinoma (ACA) and squamous cell carcinoma (SCC) samples were presented with green and blue, respectively.

Based on these two tables, 1000 feature subsets were constructed according to Eq.8. An NNA predictor was modeled for each subset and was evaluated by LOOCV. Shown in Fig.1 is the IFS curve plotted based on the data in File S3. The x-axis is the number of probes used for the classification, and the y-axis is the MCC values of classifiers evaluated by LOOCV. The maximum MCC was 0.6616 when 266 probes were utilized. With such a classifier, the prediction sensitivity, specificity and accuracy were 0.9567, 0.6452 and 0.8605, respectively. These 266 probes were regarded as the optimal biomarkers for the discrimination of two kinds of NSCLC. The information of these 266 probes were given in File S4. Shown in Fig.2 is the heatmap based on these 266 probes. It can be seen that most of the 208 ACA samples and 93 SCC samples can be distinguished.

### KEGG and GO enrichment results of CNAs genes

The KEGG pathway enrichment analysis of CNAs genes indicated that they were enriched in Wnt signaling pathway, Focal adhesion, ECM-receptor interaction and so on (Table 1). It is reported Wnt signaling pathway is activated during the carcinogenesis of NSCLC [35], and inhibition of Wnt-2-mediated signaling could induce non-small-cell lung cancer cells apoptosis [36]. Focal adhesion and ECM-receptor interaction are pathways in the biological processes interactions of cells with extracellular matrix (ECM), which play crucial roles in cell motility, cell proliferation, cell differentiation, regulation of gene expression and cell survival [37], [38]. The proteins of these pathways are up-regulated in NSCLCs [39], and take part in the activation of local invasion and distant metastasis of cancer cells [40]. As the KEGG pathway enrichment result, the GO enrichment result of these CNAs genes also shows enrichment in the terms of cell adhesion and intracellular signaling cascade. The GO enrichment result of these CNAs genes were listed in File S5.

**Table 1:** KEGG enrichment result of CNAs genes.

Pathway	KEGG ID	Your Genes (With Ann)	Your Genes (No Ann)	Genome (With Ann)	Genome (No Ann)	P-value
Wnt signaling pathway	hsa04310	6	32	141	2951	0.0077
Focal adhesion	hsa04510	7	31	227	2865	0.0204
ECM-receptor interaction	hsa04512	4	34	82	3010	0.0193

Your Genes (With Ann): The number of genes from your list with the annotation.

Your Genes (No Ann): The number of genes from your list without the annotation.

Genome (With Ann): The number of genes in the genome (excluding those in your list) with the annotation.

Genome (No Ann): The number of genes in the genome (excluding those in your list) without the annotation.

P-value: The negative logarithm of the *p* value calculated using a Fisher's exact test.

### Chromosome region enrichment result of CNAs genes

It is reported copy number gain in region 3q26 [18], [41] and in region 8p12 [42] seem to be more common in squamous histology compared with adenocarcinoma. The analysis of our result shows that including these two regions, copy number alterations of 2q34, 10p15, 18q11, 8p23, 3p21, 3q27, 22q12, Xq13, 2q36, 10p11, 10p12 also have the significance in discrimination between SCC and ACA, and deserved further researches on them (Table 2).

**Table 2:** Chromosome region enrichment result of CNAs genes.

Chromosome region	Your Genes (With Ann)	Your Genes (No Ann)	Genome (With Ann)	Genome (No Ann)	P-value
2q34	5	162	24	30139	5.09E-07
10p15	5	162	55	30108	2.04E-05
18q11	4	163	46	30117	0.0002
3q26	5	162	105	30058	0.0004
8p23	6	161	174	29989	0.0005
3p21	7	160	251	29912	0.0006
3q27	4	163	72	30091	0.0008
22q12	5	162	142	30021	0.0014
Xq13	4	163	100	30063	0.0027
2q36	3	164	51	30112	0.0033
10p11	3	164	62	30101	0.0056
10p12	3	164	63	30100	0.0058

Your Genes (With Ann): The number of genes from your list with the annotation.

Your Genes (No Ann): The number of genes from your list without the annotation.

Genome (With Ann): The number of genes in the genome (excluding those in your list) with the annotation.

Genome (No Ann): The number of genes in the genome (excluding those in your list) without the annotation.

P-value: The negative logarithm of the *p* value calculated using a Fisher's exact test.

### CNAs genes identified in this study

In this study, we identified several candidate genes corresponding to 266 CNAs probes that can be used to distinguish two kinds of NSCLC. 50 of them also has a significant correlation to the Smoking Pack-year including TP63, SOX2 and PPP2R2B (see File S4). With literature retrieval of gene function and significance comparison by p-value, we focused on 8 genes which are most probably related to distinguish ACA and SCC from each other. Among them, TP63 has been reported as a biomarker to discriminate between SCC and ACA, and it is listed top in our result. Some of other genes are reported to have different gene expression level in ACA and SCC or in patients with distinct smoking habits. In accord with the KEGG and GO enrichment result, PPP2R2B is a gene in wnt signaling pathway, while ITGA9 takes a part in focal adhesion and ECM-receptor interaction. All above illustrates that our result is biologically significant and the 8 genes may be candidate biomarkers for distinguishing ACA and SCC from each other and deserved further studies on them. Below, we will briefly discuss their relationships with NSCLC.

TP63 (Tumor protein 63) is listed top one in the optimal probe set with a CNA fold change of 0.7827 comparing ACA with SCC. It is a tumor suppressor p53 homologue and essential for p53 dependent apoptosis in response to DNA damage [43]. Mi Jin Kim et al. found P63 is a useful immunohistochemical panel in differentiating ACA from SCC of the lung with the positive rate 91% of SCC and 9% of ACA in their studies [44]. The chromosome location of TP63 is 3q27–29. Therefore, our result is coincide with former researches and TP63 may play a key role in distinguish ACA and SCC from each other.

EPHA4 (Ephrin type-A receptor 4) is related to the fourth probe in our optimal probe set with a CNA fold change of 1.0846 comparing ACA with SCC, and is a member of the Eph receptor family, the largest receptor tyrosine kinase family of transmembrane proteins with their ligands, the ephrins, affecting the growth, migration and invasion of cancer cells in culture as well as tumor growth, invasiveness, angiogenesis and metastasis in vivo [45]. Junya Fukai et al. found EphA4 promotes cell proliferation and migration through a novel EphA4-FGFR1 signaling pathway in the human glioma U251 cell line [46]. One of the Eph receptors EphA2 is reported over expression in smokers and predicts poor survival in non-small cell lung cancer [47]. A mutation in EphA2 (G391R) was identified in two of 28 squamous cell lung cancers (7%), but not in any adenocarcinomas or large-cell lung carcinomas [48]. These all indicate that EphA4 may be a candidate biomarker for distinguishing ACA and SCC from each other and deserved further studies on it.

PPP2R2B (Serine/threonine-protein phosphatase 2A 55 kDa regulatory subunit B beta isoform) is related to the fifth probe in our optimal probe set with a CNA fold change of 1.0781 comparing ACA with SCC. It is the regulatory subunit B beta isoform of PP2A, and is implicated in the negative control of cell growth and division [49]. Recently genome-wide association study (GWAS) of lung cancer in the Chinese population revealed that chromosome 5q32 (rs2895680 in PPP2R2B-STK32A-DPYSL3, P = 6.60×10−9) was lung cancer susceptibility loci and interacted with smoking dose [50]. As well as PPP2R2B is on the top of our result, the contribution of it in the NSCLC is worthy to be further elucidated.

ITGA9 (Integrin alpha-9) is related to the twelfth probe in our optimal probe set with a CNA fold change of 1.1034 comparing ACA with SCC, which belongs to the integrin family and is expressed on a wide range of cell types. It interacts with many ligands for example fibronectin, tenascin-C and ADAM12, and takes part in several processes such as cell adhesion, migration, lung development, lymphatic and venous valve development, and in wound healing [51]. ITGA9 has been found down expression in NSCLC [52], and exhibiting strong cell growth inhibition activity [53]. Statistical analysis of Alexey a. Dmitriev et al. suggested that the methylation/deletion level of ITGA9 has significant changes in ACA and SCC [53]. Our analysis presented the gene copy number of ITGA9 is dissimilar in NSCLC subtypes, implying ITGA9 as a candidate molecular to discriminate between SCC and ACA.

SOX2 (Sex-determining region Y-Box 2) is related to the nineteenth probe in our optimal probe set with a CNA fold change of 0.7790 comparing ACA with SCC, and has been reported to be differentially expressed between ACA and SCC. It is located at chromosome 3q26 and high-level amplification of SOX2 have been reported in approximately 20% of lung squamous cell carcinomas [54], [55]. SOX2 is a transcription factor controlling the expression of a number of genes involved in embryonic development and keeps neural cells undifferentiated [56]. Suppression of SOX2 in amplified SOX2 cells has greater antiproliferative effects compared with other genes on 3q26.33 including PIK3CA and TP63.

FHIT (fragile histidine triad) is related to the thirty-third probe in our optimal probe set with a CNA fold change of 1.1110 comparing ACA with SCC, and behaves in vitro as a typical diadenosine triphosphate hydrolase cleaving A-5′-PPP-5′A to yield AMP and ADP [57], but little is known about its physiological function. It is considered as a tumor suppressor in many human cancers and its restoration in Fhit-negative cancer cell lines suppresses tumorigenicity and induces apoptosis [58]. Jennifer E. Tseng el at. found that the frequency of loss of FHIT expression is related with smoking habit in Stage I Non-Small Cell Lung Cancer [59]. In the studies of Gemma Toledo et al. FHIT expression was related to tumor histology: 52 of 54 (96.3%) SCC and 20 of 44 (45.5%) ACA were negative for FHIT (P<0.0001) [60]. As SCC is closely correlated with a history of tobacco smoking [6], and our results show the copy number of FHIT is significantly lower in SCC, FHIT may be a possible biomarker for NSCLC diagnosis and would be a potential medical target for cancer therapy.

RBBP8 (Retinoblastoma-binding protein 8) is a ubiquitously expressed nuclear protein which is binding to the tumor suppressor proteins RB [61] and CtBP [62]. It is also interacting with BRCA1 [63] and is thought to regulate the functions of BRCA1 in transcriptional regulation, DNA damage repair, and G2/M cell cycle checkpoint control [64], [65]. RBBP8 is required for DNA double-strand break (DSB) resection, and thereby for recruitment of the protein kinase ATR and replication protein A to DSBs, and promotes ATR activation and homologous recombination [66]. It is reported that DNA repair components were significantly up-regulated including retinoblastoma-binding protein 8 (RBBP8), in lung SCC compared with normal lung tissue, but such up-regulation was not found in lung ACA [67]. As an essential molecular in the cell process DNA damage repair and cell cycle control, RBBP8 has the potential to be a biomarker and therapy target for NSCLC and the mechanism of its distinct expression profile in SCC and ACA deserves further study.

GPC5 (Glypican-5) is a member of the glypican gene family, which is a family of heparan sulphate proteoglycans that are linked to the exocytoplasmic surface of the plasma membrane via glycosyl phosphatidylinositol [68]. The expression level of GPC5 was significantly lower in lung adenocarcinoma tissue than in matched normal lung tissue in never smokers [69]. Yang et al. found deceased expression of GPC5 is correlated with reduced survival in ACA but not in SCC [70]. These all indicate that GPC5 may be a potential tumor suppressor gene in NSCLC, and a candidate bio-marker to discriminate between SCC and ACA.

## Conclusion

In this study, we constructed a classifier based on copy number alterations (CNA) to distinguish two subgroups of NSCLC. As a result, 266 CNA probes were selected as the best discriminators. Analysis of genes corresponding to these 266 CNA probes indicate that they were enriched in lung cancer related pathways and enriched in the chromosome regions where CNA usually occur in lung cancer. Some of these genes, such as TP63, SOX2, EPHA4, PPP2R2B, ITGA9, FHIT, RBBP8 and GPC5 are closely related to lung cancer and these candidate genes may provide clues for further research and experiment validation.

## Supporting Information

File S1 Clinical information of adenocarcinoma (ACA) and squamous cell carcinoma (SCC) samples.
(DOCX)

File S2 mRMR result for classification. This file contains two sheets. The first one is the MaxRel feature table, which ranked the top 1000 probes according to the relevance between features and class of the samples. The second one is the mRMR feature table, which ranked these 1000 probes according to the redundancy and relevance criteria.
(XLSX)

File S3 The sensitivity (Sn), specificity (Sp), accuracy (Ac), Matthews correlation coefficient (MCC) of each run of IFS for classification.
(XLSX)

File S4 The annotation of the 266 selected probes.
(XLSX)

File S5 The GO enrichment result of CNAs genes.
(XLSX)
