# Supplementary material for: Classification of Non-Small Cell Lung Cancer Based on Copy Number Alterations
Source: PLoS One. 2014 Feb 5;9(2):e88300. doi: 10.1371/journal.pone.0088300 (PMC3914971; doi:10.1371/journal.pone.0088300)
Supplement: File S1 — Clinical information of adenocarcinoma (ACA) and squamous cell carcinoma (SCC) samples. (DOCX) [file pone.0088300.s001.docx]

Table - The clinical information of ACA and SCC samples

|  | ACA | SCC |
| --- | --- | --- |
| Number of samples | 208 | 93 |
| Smoking Pack-year*  Mean (Standard Deviation) | 43.0 (35.4) | 49.7 (39.7) |
| Stage (Number of samples)** | 1 (128) : 2 (35) : 3 (11) : 4 (7) | 1 (71) : 2 (12) : 3 (7) |

*http://en.wikipedia.org/wiki/Pack-year; **only the samples with stage information were shown.

There is no significant difference between ACA and SCC on Smoking Pack-year (t test p value 0.1785).
